# A Lay Health Worker Intervention to Increase Uptake and Completion of Pulmonary Rehabilitation in Chronic Obstructive Pulmonary Disease: Assessing Fidelity of Intervention Delivery

**DOI:** 10.1080/15412555.2020.1797658

**Published:** 2020-08-17

**Authors:** Patrick White, Gill Gilworth, Viktoria McMillan, Simon Lewin, Stephanie J. C. Taylor, Alison J. Wright

**Affiliations:** a School of Population Health and Environmental Sciences, King’s College London, London, UK ;; b Norwegian Institute of Public Health, Oslo, Norway;; c Health Systems Research Unit, South African Medical Research Council, Cape Town, South Africa;; d Institute of Population Health Sciences, Bart’s and London School of Medicine and Dentistry, Queen Mary University of London, London, UK;; e Centre for Behaviour Change, Department of Clinical, Educational and Health Psychology, University College London, London, UK

**Keywords:** Community health worker, intervention fidelity, delivery style, behaviour change techniques

## Abstract

The benefits of pulmonary rehabilitation (PR) for chronic obstructive pulmonary disease (COPD) are restricted by poor uptake and completion. Lay health workers (LHWs) have been effective in improving access to treatment and services for other health conditions. We have successfully shown the feasibility of this approach in a PR setting and its acceptability to the LHWs and COPD patients. We present here the feasibility of assessment, and the fidelity of delivery of LHW support achieved for COPD patients referred for PR. LHWs, volunteer COPD patients experienced in PR, received training in the intervention including communication skills, confidentiality and behaviour change techniques (BCTs). Interactions between LHWs and patients were recorded, transcribed and coded for delivery style and BCTs. Inter-rater agreement on the coding of delivery style and BCTs was high at >84%. LHWs built rapport and communicated attentively in over 80% of interactions. LHWs most consistently delivered BCTs concerning information provision about the consequences of PR often making those consequences salient by referring to their own positive experience of PR. Social support BCTs were also used by the majority of LHWs. The use of BCTs varied between LHWs. The assessment of intervention delivery fidelity by LHWs was feasible. LHW training in the setting of PR should add emphasis to the acquisition of BCT skills relating to goal setting and action planning.

## Introduction

Pulmonary rehabilitation (PR) is effective in treating the symptoms and disability of chronic obstructive pulmonary disease (COPD) [1]. It improves health status and quality of life, it is recommended in national and international guidelines, and has been the subject of an international consensus statement [2]. Access to PR is inadequate [3]. In a UK national COPD audit only 15% of COPD patients eligible for PR were actually referred [4]. Where PR is available, its effectiveness is limited by poor uptake and completion [3, 5]. To tackle this problem, we designed an intervention in which COPD patients who had previously completed PR were trained as lay health workers (LHWs) to support newly referred patients [5]. LHWs have been shown to be effective in a range of health settings, particularly in improving uptake and adherence to proven treatments [6, 7]. We have successfully shown the feasibility of this approach in a PR setting and its acceptability to the LHWs and COPD patients [8, 9]. 

Fidelity of delivery refers to the degree to which an intervention or treatment is delivered as intended [10]. Failure to ensure fidelity compromises the validity of the evaluation of an intervention [11]. Delivery of the intervention to promote uptake and completion of PR by former patients in the role of volunteer LHWs, as intended, was likely to be challenging, as was the assessment of its fidelity. We investigated the feasibility of assessing fidelity and the level of fidelity with which the LHWs delivered the intervention to COPD patients referred for PR.

## Methods

In a feasibility study for a trial of LHW support to promote uptake and completion of PR in London, UK, we recruited and trained COPD patients experienced in PR to undertake the LHW role [9]. The volunteer LHWs attended a 3-day training programme that included communication skills; confidentiality, boundary setting and behaviour change techniques (BCTs). Behaviour change techniques are observable and replicable intervention components designed to change behaviour [12].

Fourteen BCTs were selected to address factors affecting participation in PR [13] ranging from goal setting and problem solving to provision of social support and information about the benefits of taking part in PR [12, 13]. The recruitment, selection, training and mentoring of LHWs and recruitment of COPD patient-participants have been described elsewhere [8]. Written informed consent was obtained from all participants.

LHWs were provided with smartphones to record interactions (telephone and face-to-face contacts) with patient-participants. The feasibility of using this method to assess delivery fidelity was evaluated by measuring the proportion of interactions that were recorded and could be transcribed and entered into the analysis. Each series of interactions between an LHW and patient pair was transcribed as a single transcript. Intervention delivery fidelity was assessed by analysing delivery style and BCT delivery in a sample of transcripts [9]. Analysis was based on a coding framework developed and piloted from the transcribed interactions of 3 LHW-patient pairs by PW, AW and GG. Transcripts were coded independently by two coders and discrepancies in coding then resolved by discussion. The amended framework was tested by the three coders on a further three LHW–patient-participant pairs in the pilot stage.

The main analysis was carried out by AW and VMcM on a sample of transcripts consisting of two pairings for each LHW. The two LHW-patient transcripts were selected for analysis of the delivery style and behaviour change techniques used by each LHW. The first transcript selected was of the first patient-participant supported by each LHW. The second transcript was of a patient-participant supported at the half-way point of the LHW’s work with patients. If there was no available recording for the identified pairing, the next available patient-participant supported by the LHW was selected for analysis instead. Using this method, it was hoped to avoid omission bias, and also to avoid the risk of selection bias (i.e. choice of the ‘best’ pairings or best interaction sets for a pairing).

Before coding the transcripts, the coders discussed the definitions of delivery style and behaviour change technique laid out in the research protocol in order to limit disparities in interpretation. The coding was carried out independently and disagreements were noted.

Inter-rater agreement was assessed by the proportion of all instances of delivery style and BCTs that were identified by both coders [14]. This study follows the ethical principles of the Declaration of Helsinki. Ethical approval was provided by NRES Committee, London – Westminster. REC reference 14/LO/2313

## Results

Sixty-six COPD patients were supported by 12 LHWs: 5.5 patients per LHW (range 3–8). LHW demographic data are shown in Table 1. There was a gap of up to 3 months between the training of LHWs and the first recruitment of patients due to initial low response to invitation by patient-participants [9]. Recordings were made by LHWs with 60 patient-participants. Recordings were not available for six LHW-patient pairs due to problems with equipment. One LHW lost their phone for three weeks. 360 interactions were reported by the LHWs of which 329 interactions were recorded and transcribed. Some pairs had frequent and prolonged contact over a 2–3-month period. LHW-patient pairs had 5.4 interactions on average; in two pairs there were 20 or more interactions.

**Table 1.** Lay health workers’ age, gender, patient-participants supported, interactions^a^ undertaken and interactions transcribed.

LHW	Age	Sex	Patient-participants supported	Number of interactions with all patient-participants (mean per patient)	Number of interactions transcribed (percent)
A	55–59	M	3	9 (3)	7 (78%)
B	75–79	M	4	14 (3.5)	14 (100%)
C	65–69	M	4	25 (6.3)	19 (76%)
D	65–69	F	8	85 (10.6)	84 (99%)
E	55–59	F	7	15 (2.1)	10 (67%)
F	60–64	F	4	10 (2.5)	8 (80%)
G	75–79	M	7	17 (2.4)	14 (82%)
H	55–59	F	8	60 (7.5)	58 (97%)
I	65–69	F	7	61 (8.7)	54 (89%)
J	70–74	M	6	28 (4.7)	18 (64%)
K	65–69	M	4	10 (2.5)	5 (50%)
L	79–79	F	4	26 (6.5)	23 (88%)

^a^Interactions include telephone and face to face encounters.

Twenty-four transcripts were coded, two for each LHW, 40% of all transcripts. One hundred twenty-five interactions were included in the 24 transcripts, 39% of all interactions. Three hundred and fifty-four instances of behaviour change techniques were identified with coder agreement in 84%. Sixty-five instances of delivery style were identified with coder agreement in 89%.

The five components of delivery style assessed in the coding framework and their use in the transcripts analysed are shown in Table 2. Some of the components were utilised by the majority of LHWs: for example, evidence of LHWs attempt to build rapport with the patients they were supporting was found in 20 (83%) of the 24 transcripts coded. In contrast, the eliciting of barriers and facilitators to PR attendance, which was intended to be part of tailoring the intervention to patient-participants’ needs, was used by fewer LHWs (38%).

**Table 2.** Five components of delivery style taught to lay-health workers and the frequency with which they were coded in transcripts of the recorded meetings of 24 selected LHW-patient pairs.

Component of delivery style	Number of selected LHW-patient pairings where this component was coded (%)	Examples from transcripts
LHW makes attempts to build rapport by finding common ground (in terms of illness experiences, but also other aspects of life)	20 (83%)	LHW: ‘*Do you know what? The same thing happened to me before I got on the PR programme’.* (LHW I/Pt 46)
LHW asks open questions	15 (63%)	LHW: ‘…*so how did you find the classes*?’ (LHW A/Pt 37) LHW: ‘*How did you get on?*’ (LHW I/Pt 46)
LHW tries to elicit barriers and facilitators to PR relevant to the participant	9 (38%)	LHW: ‘*Is there any reason why…?*’ (LHW E/Pt 17) LHW: ‘*I know none of us like to go to hospital but you’re quite happy getting there and sorting things out?’* (LHW C/Pt 27)
LHW responds flexibly to issues, facilitators and barriers important to the participant	10 (42%)	Patient: ‘*He’s booked an appointment for Monday 4^th^ April, which is one of my days I should be at*…’ LHW: ‘*That’s all right. If you let them know, they’ll put that down and add it onto the end of your programme’*. (LHW I/Pt 46)
LHW is attentive and clearly interested in and responding to the patient’s communication, both in terms of its content and feeling	21 (88%)	LHW: ‘*Great, well I hope you find it OK and I’ll ring you again next week if that’s OK, just to see how you’re going on?’* (LHW A/Pt 37) LHW: ‘*Or would you rather me ring you when you come back*?’ (LHWB/Pt 1)

The rates of use of the BCTs are shown in Table 3. Details of the BCTs with definitions and examples from the transcripts are available in Supporting Information Table S1 [12]. LHWs frequently used BCTs to provide information about the consequences of attending PR, often making those consequences salient by referring to their own positive experience of PR. Social support BCTs were also used by the majority of LHWs. BCTs relating to goal setting and action planning were rarely used.

**Table 3.** Number of interactions^a^ in selected lay health worker (LHW)-patient pairs and frequency of use of behaviour change techniques (BCTs) by lay health workers in those pairs.

Lay health worker	A	B	C	D	E	F	G	H	I	J	K	L
Number of interactions^a^ in two selected LHW-patient pairs for each LHW	4	10	10	18	6	4	6	16	25	12	5	9
**Behaviour change techniques**	**Frequency of use of BCT**											
Goal setting (behaviour)	1	0	0	0	0	0	0	0	0	1	0	0
Problem solving	1	0	0	1	0	0	0	0	0	0	0	0
Goal setting (outcome)	0	0	0	0	0	0	0	0	0	1	0	1
Action planning	Not used in any LHW-patient participant pair											
Social support (unspecified)	3	8	0	5	0	3	0	5	10	12	1	5
Social support (practical)	0	0	4	7	1	0	0	0	2	7	0	1
Social support (emotional)	0	0	0	3	0	1	0	2	0	6	0	1
Information about health consequences	4	5	7	18	0	2	0	2	11	11	0	3
Salience of consequences	5	15	9	16	1	7	3	5	23	23	0	2
Information about social and environmental consequences	2	1	6	9	1	2	2	6	14	23	0	2
Information about emotional consequences	0	0	2	3	0	3	0	2	5	5	0	1
Social comparison	Not used in any LHW-patient participant pair											
Information about others’ approval	0	0	0	0	0	0	0	2	0	0	0	0
Social reward	0	0	0	2	0	0	0	0	7	2	0	0

^a^Interaction includes telephone and face to face encounter.

## Discussion

We have demonstrated the feasibility of evaluating the fidelity with which trained volunteer LHWs, (COPD patients who had previously completed PR themselves), deliver BCT-based support to COPD patients referred to PR. This is a key issue in designing a definitive trial of the LHW intervention in PR services. The feasibility of recruiting and training such LHWs and the acceptability of the intervention to patients have already been reported [8].

The LHWs, successfully tried to build rapport with and respond attentively to the patients they supported. The most used BCT, ‘Salience of Consequences’, emphasising and making memorable the consequences of a behaviour, was one the LHWs were uniquely placed to use to promote PR, illustrating the information they provided about PR’s benefits with vivid examples from their personal experiences. The LHWs’ support was intended to be tailored to the barriers and facilitators to PR attendance and completion most relevant to each patient-participant, but LHWs did not always elicit these barriers/facilitators to enable this personalisation.

The variable use by LHWs of the elements of delivery style and of the BCTs may represent a gap in the effectiveness of the LHW training. Better fidelity of delivery may be achieved by revising the training to place greater emphasis on the elements that were least used by LHWs and ensuring that the eliciting of barriers and facilitators to PR are given more focus in order to personalise support. The pace of learning differed between LHWs. Our qualitative data showed that LHWs would have been willing to undertake more training [8]. A training package responding to differences in learning pace and providing targeted reinforcement of key areas should be considered. The gap of 3 months between the training of LHWs and the recruitment of patient-participants may have led to attenuation of the skills taught in training. LHWs were provided with monthly mentoring and peer support. In future, this could provide additional opportunity to promote LHWs delivering the more complex elements of the intervention consistently [8].

The strengths of this study include assessing fidelity of delivery in terms of both delivery style and intervention content (BCTs). The coding scheme was developed and refined by a multidisciplinary team, and inter-rater agreement, >84%, across coded transcripts was well above the 75% stated in the literature as being the threshold for high agreement [14, 15].

There are additional elements of treatment fidelity that were not assessed in this study [9]. These include delivery of the training content to the LHWs as intended by the trainer and assessment of the acquisition of the relevant skills by LHWs. These elements would help to determine whether lower use of some BCTs was due to inadequate attention to them in training or to difficulty in learning those skills. Uncertainty before the study about the acceptability of this novel intervention to LHWs with COPD themselves, had led to a study design that limited as much as possible the training burden on the new LHWs [16]. Their willingness to consider undertaking more training supports the acceptability of assessing these elements of fidelity [8].

We have found little evidence in the COPD literature, or in that of other chronic diseases, of the evaluation of fidelity of an intervention based on a formal course of training, and delivered by volunteer patients with the same disease and experience of the treatment. There are, nonetheless, many examples in low, middle and high income countries of LHWs who have the disease and experience of the treatment which they have been recruited to promote [7, 17, 18].

In conclusion, this article shows that assessing fidelity of delivery of a LHW intervention to promote PR completion is feasible. We found appropriateness of delivery style was high in LHW-patient-participant interactions. Future LHW training should add emphasis to tailoring support to individual PR patients’ needs.

## Supplementary Material

Supplemental Material
