# Supplementary material for: A Lay Health Worker Intervention to Increase Uptake and Completion of Pulmonary Rehabilitation in Chronic Obstructive Pulmonary Disease: Assessing Fidelity of Intervention Delivery
Source: COPD. 2020 Aug 17;17(5):557–61. doi: 10.1080/15412555.2020.1797658 (PMC9397129; doi:10.1080/15412555.2020.1797658)
Supplement: Supplemental Material [file ICOP_A_1797658_SM0549.pdf]

Table S1 Behaviour change techniques [12] identified as possible LHW responses to reported obstacles to take-up or completion of pulmonary rehabilitation.

BCT code	BCT name	BCT definition	Example from transcripts
1.1	Goal setting (behaviour)	'Set or agree on a goal defined in terms of the behaviour to be achieved'	Patient: "*...having missed a couple I expect I'll have gone backwards a bit again*."<br><br>LHW: "*Don't worry about that, how about we set a goal that you start back again next Friday?*"<br><br>(LHW A/Pt 97)
1.2	Problem solving	'Analyse, or prompt the person to analyse, factors influencing the behaviour and generate or select strategies that include overcoming barriers and/or increasing facilitators'	Patient: "*So hopefully I'll be able to do that on Thursday.*"<br><br>LHW: "*That's good, that's lovely. At least try and if not, perhaps they can arrange transport for you*." (LHW D/Pt 9)
1.3	Goal setting (outcome)	'Set or agree on a goal defined in terms of a positive **outcome** of wanted behaviour'	LHW: "*I mean that programme that you're doing, um, you're enjoying, you know, you like it and you want to get the gym membership afterwards which I totally agree I think is, is really a great idea…"*(LHWL/Pt 22)
1.4	Action planning	'Prompt detailed planning of performance of the behaviour (must include at least one of context, frequency, duration and intensity). Context may be environmental (physical or social) or internal (physical, emotional or cognitive)'	Not used in any LHW-patient participant pair
3.1	Social support (unspecified)	'Advise on, arrange or provide social support or non-contingent praise or reward for performance of the behaviour. It includes encouragement and counselling, but only when it is directed at the **behaviour**'	LHW: "*I'll be very happy to be of any help, to chat with you and if need be, to come and see you*."<br><br>(LHW K/Pt 32)
3.2	Social support (practical)	'Advise on, arrange, or provide **practical** help *(e.g. from friends, relatives, colleagues, or staff)* for performance of the behaviour'	LHW: "*Plan, yeah. But you seem, do you want me to meet before you go there or are you okay going?*" (LHW E/Pt 11)

3.3	Social support (emotional)	'Advise on, arrange, or provide **emotional** social support for performance of the behaviour'	LHW: "*Oh crikey. Well you know, I'm here if you want to talk. I don't mind phoning you up and chatting to you now and again if that will help you in any way?*" (LHW F/Pt 48)
5.1	Information about health consequences	'Provide information (e.g. written, verbal, visual) about health consequences of performing the behaviour'	LHW: "*It's, um, I found before I went to the rehabilitation I could only walk 50, 60 yards, I had to stop for breath and that sort of thing and although I still have bad days, er, I'm so glad that I went to rehabilitation because they taught me breathing and the exercises to do and…*"(LHW B/Pt 1)
5.2	Salience of consequences	'Use methods specifically designed to **emphasise** the consequences of performing the behaviour with the aim of making them more memorable (goes beyond informing about consequences)'	LHW: "*Oh you do, what I did (pt name) when I did mine at (hospital) I had the, um, the exercises first and then the education bit after.*"<br><br>Patient: "*Oh yeah, of course. Yeah, you was at (hospital name)?*"<br><br>LHW: "*Yeah, I was at, yeah, I was at (same hospital name), yeah. As I say we had the, you know, the exercises and then we sat down for the education, had a cup of tea and a biscuit, which was quite nice, yeah.*" (LHW H/Pt 5)
5.3	Information about social and environmental consequences	'Provide information (e.g. written, verbal, visual) about social and environmental consequences of performing the behaviour'	LHW: "*Well this is it, you're socialising as well because you're meeting different people.*" (LHW I/Pt 46)
5.6	Information about emotional consequences	'Provide information (e.g. written, verbal, visual) about emotional consequences of performing the behaviour'	LHW: "*I used to live in a small envelope. That's what I thought I was capable of. What the PR sessions taught me was that I was actually capable of a lot more, so now my limits are bigger. Yes, I can do this. I used to try and avoid getting breathless but one of the things they teach, don't be frightened of getting breathless. If you can walk a little bit faster*" (LHW C/Pt 17)
6.2	Social comparison	'Draw attention to others' performance to allow	Not used in any LHW-patient participant pair

		comparison with the person's own performance'	
6.3	Information about others' approval	'Provide information about what other people think about the behaviour. The information clarifies whether others will like, approve or disapprove of what the person is doing or will do'	LHW: "*It makes a hell of a difference, yeah, and you'll be able to run up the bleeding stars soon girl, with the Hoover…He'll be well pleased, the old man, 'get up there!"* (LHW J/Pt 51)
10.4	Social reward	'Arrange verbal or non-verbal reward if and only if there ***has been*** effort and/or progress in performing the behaviour'	LHW: "*I'm really pleased for you honestly, just keep it up*." (LHW I/Pt 2)
